# Microplastics in the rhizosphere: unraveling plant–microbe–soil interactions and consequences for crop resilience

**DOI:** 10.1080/15592324.2026.2678701

**Published:** 2026-05-23

**Authors:** Taiba Saeed, Tanveer Alam Khan, Mohammad Yusuf, Andrej Bajguz

**Affiliations:** a Department of Biosciences, Integral University, Lucknow, Uttar Pradesh, India; b Department of Biology, College of Science, United Arab Emirates University, Al Ain, UAE; c Department of Biology and Plant Ecology, Faculty of Biology, University of Bialystok, Bialystok, Poland

**Keywords:** Microplastic pollution, agricultural sustainability, plant-soil interactions, ecotoxicological stressors, climate-pollutant interactions

## Abstract

Microplastics (MPs), plastic particles smaller than 5 mm, are increasingly recognized as pervasive pollutants in terrestrial ecosystems, especially agricultural soils, which serve as long-term sinks. While early research prioritized aquatic environments, recent studies underscore the diverse pathways through which MPs infiltrate soils, via plastic mulching, wastewater irrigation, sewage sludge, compost, and atmospheric deposition. This review provides a comprehensive overview of emerging insights into MPs-plant-microbe interactions within soil systems, emphasizing both their complex ecological effects and key knowledge gaps. The main objective of this review is to consolidate current evidence on how MPs affect plant physiology and soil microbial dynamics, and to highlight methodological limitations impeding progress in this field. MPs exhibit variable but often detrimental effects on plant health, including delayed germination, inhibited growth, impaired photosynthesis, and disrupted nutrient uptake. These outcomes are largely driven by physical blockage, chemical leaching, and oxidative stress, and are influenced by MPs characteristics (polymer type, shape, concentration) and plant species traits. Interestingly, low MPs levels may occasionally improve root biomass through enhanced soil aeration and water retention, reflecting the context-dependent nature of MPs impacts. Crucially, MPs alter soil microbial communities, reducing beneficial microbes, promoting pathogens, and interfering with enzymatic functions, thereby indirectly undermining soil fertility and crop productivity. Disruption of symbiotic relationships, such as mycorrhizal associations, further compounds ecological stress. This review also identifies a pressing need for standardized MPs detection and toxicity assessment protocols. Advancing analytical tools and ecologically relevant models is essential for uncovering plant molecular responses and supporting sustainable agriculture in MPs-contaminated environments.

## 1. Introduction

Microplastics (MPs), defined as plastic particles smaller than 5 mm, have emerged as a pervasive environmental contaminant, raising growing concerns about their ecological and biological impacts.^1^ Terrestrial environments, particularly agricultural soils, are susceptible to MPs contamination.^2^ These particles originate from diverse sources, including plastic mulch degradation, wastewater irrigation, sewage sludge application, compost inputs, and atmospheric deposition. Due to their physicochemical persistence, MPs can accumulate in soils and alter crucial ecosystem processes.^3^ ^,^ ^4^ Soils are foundational to terrestrial ecosystems, particularly in agroecosystems where they support plant growth and mediate critical biogeochemical cycles. MPs in soil can influence structural properties such as porosity, water retention, and aggregate stability, with downstream effects on nutrient cycling and plant productivity.^2^ ^,^ ^3^ However, the rhizosphere, the narrow zone of soil directly influenced by plant roots, is a critical interface where plant-microbe-soil interactions occur. This dynamic region is characterized by high microbial activity, driven by root exudates that provide carbon and energy sources for diverse microbial communities.^5^ The presence of MPs in the rhizosphere can disrupt these finely tuned interactions, with potential consequences for plant growth, stress tolerance, and overall ecosystem functioning. Understanding how MPs affect rhizosphere processes is therefore essential for predicting their broader ecological impacts and developing strategies to mitigate their effects in agricultural systems.

This review demonstrates that MPs affect plant health not only through direct physical and chemical stress but also indirectly by altering soil physicochemical properties and disrupting microbial community structure and function. By integrating current evidence on MPs sources, distribution, and impacts in the rhizosphere, this review aims to provide a comprehensive understanding of the complex interactions between MPs, plants, and soil microbes. It also highlights critical research needs, including the development of advanced analytical techniques, the establishment of standardized testing protocols, and the integration of molecular and ecological approaches to uncover the mechanisms of MPs toxicity. Ultimately, this review seeks to inform sustainable agricultural practices and policy decisions aimed at reducing MPs pollution and protecting soil health in the face of increasing plastic use and environmental contamination.

## 2. Sources and pathways of MPs in agricultural soils

Agricultural soils are exposed to MPs through multiple pathways, each contributing to the accumulation of these persistent pollutants. Understanding these sources is crucial for developing effective mitigation strategies and assessing the long-term risks associated with MPs contamination in agroecosystems.

### 2.1. Plastic mulching

Plastic mulch films, widely used in agriculture to enhance crop yields by regulating soil temperature, conserving moisture, and suppressing weeds, are a major source of MPs in agricultural soils.^6^ These films, typically made from polyethylene (PE), are prone to fragmentation due to UV radiation, mechanical stress, and microbial degradation, leading to the release of MPs into the soil.^7^ Studies have shown that plastic mulch can contribute up to 120 kg of MPs per hectare annually, with concentrations increasing over time in fields where mulch is repeatedly applied.^6^ The persistence of these particles in soil, combined with their potential to leach additives such as plasticizers and stabilizers, raises concerns about their long-term ecological impacts.^8^ Recent research has demonstrated that both conventional and biodegradable mulching films can significantly affect soil properties, microbial activities, and plant performance, with effects varying across different pedoclimatic conditions.^9,10,11,12^

### 2.2. Wastewater irrigation and sewage sludge

Wastewater irrigation and the application of sewage sludge as fertilizer are significant pathways for MPs entry into agricultural soils. Wastewater treatment plants are not designed to remove MPs, and effluents can contain high concentrations of these particles, which are then transferred to soils during irrigation.^13^ Similarly, sewage nutrients are rich in organic matter and nutrients, often containing MPs derived from household and industrial sources.^14^ Studies have reported MPs concentrations in sewage sludge ranging from 1,000 to 24,000 particles per kilogram, with PE and polypropylene (PP) being the most common polymer types.^15^ The application of sewage sludge to agricultural fields can introduce substantial quantities of MPs, with estimates suggesting that up to 63,000 to 430,000 tons of MPs are added to European soil annually through this practice.^13^

### 2.3. Compost and organic amendments

Compost and other organic amendments, often derived from municipal solid waste or agricultural residues, can also be sources of MPs in agricultural soils. Plastic contaminants in compost feedstocks, such as plastic bags, packaging materials, and synthetic fibers, can fragment during the composting process, leading to the formation of MPs.^16^ Studies have detected MPs in compost at concentrations ranging from 14 to 895 particles per kilogram, with PE and PP being the predominant polymer types.^16^ The application of MPs-contaminated compost to agricultural fields can contribute to the accumulation of these particles in soil, with potential implications for soil health and crop productivity.

### 2.4. Atmospheric deposition

Atmospheric deposition is an emerging pathway for MPs entry into agricultural soils, particularly in regions with high levels of air pollution or proximity to urban and industrial areas. MPs can be transported through the atmosphere as airborne particles, originating from sources such as tire wear, textile fibers, and plastic waste.^17^ Studies have reported MPs deposition rates ranging from 3 to 10 particles per square meter per day in urban areas, with smaller particles (<50 μm) being more readily transported over long distances.^17^ While the contribution of atmospheric deposition to total MPs loads in agricultural soils is not yet fully quantified, it represents a potentially significant and widespread source of contamination, particularly in regions with intensive agricultural activity and high levels of plastic use.

### 2.5. Other sources

Other sources of MPs in agricultural soils include the use of plastic-coated fertilizers, pesticides, and seed coatings, as well as the degradation of agricultural equipment and infrastructure (e.g., irrigation pipes, greenhouse films).^8^

## 3. Types and classification of MPs: biodegradable MPs in agricultural soils

**Figure 1.**
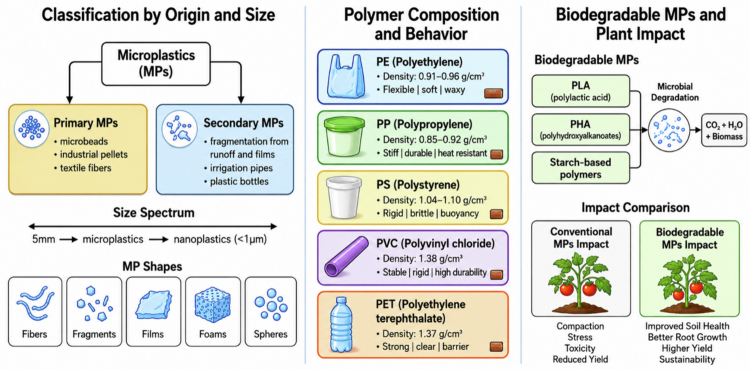
Classification, composition, and environmental implications of MPs. MPs are classified into primary MPs, including microbeads, industrial pellets, and textile fibers, and secondary MPs generated through the degradation and fragmentation of larger plastic materials such as irrigation pipes, plastic films, and bottles. The size continuum ranges from microplastics to nanoplastics (<1 μm), with common morphologies including fibers, fragments, films, foams, and spheres. Major polymer types, including polyethylene (PE), polypropylene (PP), polystyrene (PS), polyvinyl chloride (PVC), and polyethylene terephthalate (PET), differ in density, rigidity, durability, buoyancy, and environmental behavior. Biodegradable MPs, such as polylactic acid (PLA), polyhydroxyalkanoates (PHA), and starch-based polymers, undergo microbial degradation, producing CO₂, H₂O, and biomass as end products. Compared with conventional MPs, biodegradable MPs generally exert fewer adverse effects on soil and plant systems by improving soil health, enhancing root growth, reducing toxicity, and supporting higher crop productivity.

MPs are classified based on their origin, size, shape, and polymer composition, each of which influences their environmental behavior and ecological impacts. Understanding these classifications is essential for assessing the risks associated with MPs contamination and developing targeted mitigation strategies (Figure 1).

### 3.1. Primary and secondary MPs

MPs are broadly categorized into primary and secondary types based on their origin (Figure 1). Primary MPs are manufactured at microscopic sizes for specific applications, such as microbeads in personal care products, plastic pellets used in industrial processes, and abrasives in cleaning products.^18^ Secondary MPs, on the other hand, result from the fragmentation of larger plastic items due to physical, chemical, and biological degradation processes.^19^ In agricultural soils, secondary MPs are more prevalent, originating from the breakdown of plastic mulch films, irrigation pipes, and other plastic materials used in farming practices.^6^

### 3.2. Size and shape

The size and shape of MPs are critical factors that determine their mobility, bioavailability, and toxicity (Figure 1). MPs are typically defined as particles smaller than 5 mm, but they can range from a few micrometers to several millimeters in size.^20^ Smaller particles, particularly those in the nanoplastic range (<1 μm), are of particular concern due to their ability to penetrate biological membranes and accumulate in plant and animal tissues.^21^ The shape of MPs also varies widely, including fibers, fragments, films, foams, and spheres, each with distinct physical and chemical properties.^22^ Fibers, for example, are more likely to become entangled in soil aggregates and root systems, while spherical particles may be more readily transported through soil pores.^8^

### 3.3. Polymer composition

MPs are composed of a wide range of synthetic polymers, each with unique chemical properties and environmental behaviors. The most common polymer types found in agricultural soils include PE, PP, PS, PVC, and PET.^20^ These polymers differ in their density, hydrophobicity, and susceptibility to degradation, which influence their persistence and mobility in soil.^19^ For example, PE and PP are less dense than water and tend to accumulate at the soil surface, while PVC and PET are denser and may migrate deeper into the soil profile.^8^

### 3.4. Biodegradable MPs

Biodegradable plastics, designed to degrade more rapidly than conventional plastics, have been promoted as a sustainable alternative for agricultural applications, particularly for mulch films.^23^ These materials are typically composed of bio-based polymers such as polylactic acid (PLA), polyhydroxyalkanoates (PHA), or starch-based blends, which are intended to break down into water, carbon dioxide, and biomass under specific environmental conditions.^24^ However, the degradation of biodegradable plastics in soil is highly variable and depends on factors such as temperature, moisture, microbial activity, and polymer composition.^24^ Studies have shown that biodegradable plastics can persist in soil for extended periods, particularly under suboptimal conditions, and may fragment into MPs before complete degradation occurs. ^25^Recent studies have shown that biodegradable MPs can induce significant changes in plant defense mechanisms and affect growth traits, with effects comparable to or sometimes exceeding those of conventional MPs^11^ ^,^ ^26^ (Figure 1).

## 4. The role of bioturbation in MP movement in soil

Bioturbation, the physical mixing of soil by organisms such as earthworms, arthropods, and plant roots, plays a critical role in the distribution and transport of MPs within soil profiles.^27^ This process can facilitate the vertical and horizontal movement of MPs, influencing their bioavailability and potential for uptake by plants and soil organisms.

### 4.1. Earthworms and MPs transport

Earthworms are among the most important bioturbators in agricultural soils, contributing to soil structure, nutrient cycling, and organic matter decomposition.^28^ These organisms ingest soil particles, including MPs, as they burrow through the soil, and their feeding and burrowing activities can significantly alter the distribution of MPs in the soil profile.^27^ Studies have shown that earthworms can transport MPs from the soil surface to deeper layers, with some species capable of moving particles up to 30 cm below the surface.^27^ This vertical transport can increase the persistence of MPs in soil and enhance their potential for interaction with plant roots and soil microbes.

In addition to their role in MPs transport, earthworms can also influence the physical and chemical properties of MPs. For example, the passage of MPs through the earthworm gut can alter their surface characteristics, potentially increasing their bioavailability or toxicity.^29^ Moreover, earthworms can fragment larger plastic particles into smaller MPs through mechanical grinding in their gizzards, further contributing to the proliferation of these particles in soil.^29^ Recent research has shown that earthworms can significantly modify the effects of mulching film MPs on soil properties and plant growth, with their presence altering microbial activities and nutrient dynamics.^10^

### 4.2. Arthropods and other soil fauna

Arthropods, including insects, mites, and springtails, also contribute to bioturbation and MPs movement in soil. These organisms can transport MPs through their feeding and burrowing activities, as well as through the construction of nests and burrows.^30^ For example, ants have been shown to transport MPs from the soil surface to their nests, where they can accumulate in high concentrations.^31^ Similarly, termites and other wood-feeding insects can ingest MPs present in decaying plant material, potentially facilitating their incorporation into the soil food web.^30^ Other soil fauna, such as nematodes and protozoa, may also interact with MPs, although their role in MPs transport is less well understood. These organisms can influence the distribution of MPs through their feeding activities and by altering soil structure and porosity.^32^ For example, nematodes can create channels in the soil as they move, which may facilitate the movement of MPs through the soil profile.^32^

### 4.3. Plant roots and rhizosphere processes

Plant roots also contribute to bioturbation and MPs movement in soil through their growth and exudation activities. As roots penetrate the soil, they can physically displace MPs, altering their distribution and bioavailability.^33^ Root exudates, which include organic acids, sugars, and amino acids, can also influence the behavior of MPs by altering their surface properties or promoting microbial colonization.^5^ For example, root exudates can enhance the aggregation of MPs, reducing their mobility and bioavailability, or they can facilitate the breakdown of MPs by stimulating microbial activity.^32^

## 5. Physicochemical interactions of MPs with soil components

The physicochemical properties of MPs and their interactions with soil components play a critical role in determining their environmental behavior and ecological impacts. These interactions influence the mobility, bioavailability, and toxicity of MPs, as well as their effects on soil structure, water retention, and nutrient cycling.

### 5.1. Sorption and desorption

MPs can sorb a wide range of organic and inorganic contaminants from the soil environment, including heavy metals, pesticides, and polycyclic aromatic hydrocarbons (PAHs).^34^ This sorption is driven by the hydrophobic nature of many plastic polymers, which allows them to accumulate lipophilic compounds on their surfaces.^35^ The sorption capacity of MPs depends on factors such as polymer type, particle size, surface area, and the presence of additives or weathering products.^34^ For example, weathered MPs with increased surface roughness and porosity tend to have higher sorption capacities than pristine particles.^35^ The sorption of contaminants to MPs can have both positive and negative effects on soil health and plant productivity. On one hand, MPs can act as sinks for pollutants, reducing their bioavailability and toxicity to plants and soil organisms.^34^ On the other hand, MPs can serve as vectors for the transport and release of contaminants, potentially increasing their exposure to plants and soil microbes.^35^ The desorption of contaminants from MPs is influenced by factors such as pH, temperature, and the presence of organic matter, and can result in the release of toxic compounds into the soil environment.^35^

### 5.2. Effects on soil structure and aggregation

MPs can influence soil structure and aggregation by altering the physical properties of soil particles and their interactions with organic matter and microbial communities. Studies have shown that MPs can reduce soil bulk density and increase porosity, potentially enhancing water infiltration and root penetration.^36^ However, these effects are highly variable and depend on factors such as MPs concentration, particle size, and soil type.^36^ For example, high concentrations of MPs can disrupt soil aggregation by interfering with the binding of soil particles by organic matter and microbial exopolysaccharides, leading to reduced soil stability and increased erosion.^8^

The presence of MPs in soil can also affect water retention and hydraulic conductivity. Some studies have reported that MPs can increase water-holding capacity by creating additional pore spaces, while others have found that they can reduce water retention by disrupting soil structure and aggregation.^36^ These effects are likely to vary depending on the type and concentration of MPs, as well as the physical and chemical properties of the soil.^36^

### 5.3. Nutrient cycling and availability

MPs can influence nutrient cycling and availability in soil by altering the physical and chemical properties of the soil environment and by interacting with soil microbes and plant roots. For example, MPs can sorb nutrients such as nitrogen and phosphorus, reducing their bioavailability to plants and microbes.^37^ This sorption can be particularly pronounced for charged nutrients, such as ammonium and phosphate, which can bind to the surfaces of weathered MPs with increased surface charge.^37^ In addition to their direct effects on nutrient availability, MPs can also influence nutrient cycling by altering microbial community composition and activity. Studies have shown that MPs can inhibit the activity of key enzymes involved in nutrient transformation, such as urease, phosphatase, and dehydrogenase, potentially reducing the rates of nitrogen mineralization and phosphorus solubilization.^32^ These effects can have cascading impacts on plant productivity and soil fertility, particularly in nutrient-limited systems.^32^

## 6. Analytical techniques for MPs detection in soil

The detection and quantification of MPs in soil are challenging due to the complexity of the soil matrix, the wide range of MPs sizes and shapes, and the lack of standardized methodologies. Advances in analytical techniques are essential for improving our understanding of MPs contamination in agricultural soils and for assessing the risks associated with these pollutants.

### 6.1. Sample preparation and extraction

The first step in MPs analysis is the extraction of MPs from the soil matrix, which typically involves a combination of physical and chemical methods. Physical methods, such as sieving and density separation, are used to separate MPs from soil particles based on their size and density.^20^ Density separation is particularly effective for isolating MPs, as most plastic polymers have densities lower than that of soil minerals.^20^ Common density separation solutions include sodium chloride (NaCl), zinc chloride (ZnCl₂), and sodium polytungstate (SPT), each with different densities and efficiencies.^20^

Chemical methods, such as digestion with hydrogen peroxide (H₂O₂) or enzymatic treatments, are used to remove organic matter and other interfering substances from the soil sample.^20^ These methods can improve the purity of the MPs extract and facilitate subsequent analysis, but they must be carefully optimized to avoid damaging or altering the MPs.^20^ For example, prolonged exposure to strong oxidizing agents can cause fragmentation or chemical modification of MPs, leading to underestimation of MPs concentrations.^20^

### 6.2. Visual identification and microscopy

Visual identification and microscopy are commonly used for the initial screening and characterization of MPs in soil samples. Stereomicroscopy allows for the examination of MPs at low magnifications (10-50×), enabling the identification of larger particles (>500 μm) based on their shape, color, and surface characteristics.^22^ However, visual identification is subjective and prone to errors, particularly for smaller particles or those with irregular shapes.^22^

Scanning electron microscopy (SEM) provides higher resolution imaging of MPs, allowing for the detailed examination of surface morphology and the identification of weathering features such as cracks, pits, and biofilms.^22^ SEM can also be coupled with energy-dispersive X-ray spectroscopy (EDS) to provide elemental composition data, which can aid in the identification of polymer types and additives.^22^ However, SEM is time-consuming and requires specialized equipment and expertise, limiting its applicability for routine MPs analysis.^22^

### 6.3. Spectroscopic techniques

Spectroscopic techniques, such as Fourier-transform infrared spectroscopy (FTIR) and Raman spectroscopy, are widely used for the chemical identification of MPs in soil samples. FTIR measures the absorption of infrared light by chemical bonds in the polymer, producing a characteristic spectrum that can be used to identify the polymer type.^20^ FTIR can be performed in transmission, reflectance, or attenuated total reflectance (ATR) modes, each with different sensitivities and sample requirements.^20^ Micro-FTIR, which combines FTIR with microscopy, allows for the analysis of individual particles as small as 10–20 μm, making it suitable for the detection of smaller MPs.^20^ Raman spectroscopy measures the inelastic scattering of light by molecular vibrations, providing complementary information to FTIR.^20^ Raman spectroscopy is particularly useful for the identification of polymers with weak infrared absorption, such as PE and PP, and can be used to analyze particles as small as 1 μm.^20^ However, Raman spectroscopy is susceptible to fluorescence interference from organic matter and other contaminants, which can obscure the Raman signal and complicate data interpretation.^20^

### 6.4. Advanced imaging techniques

Advanced imaging techniques, such as micro-computed tomography (µ-CT) and fluorescence microscopy, are emerging tools for the detection and characterization of MPs in soil. µ-CT uses X-ray imaging to create three-dimensional reconstructions of soil samples, allowing for the visualization of MPs within the soil matrix without the need for extraction or sample preparation.^38^ This technique is particularly useful for studying the spatial distribution of MPs in soil and their interactions with soil structure and pore networks.^38^ However, µ-CT is limited by its relatively low resolution (typically 1–10 μm) and the difficulty of distinguishing MPs from other soil components with similar densities.^38^

Fluorescence microscopy, combined with fluorescent dyes or tracers, can be used to visualize MPs in soil and plant tissues.^38^ This technique is particularly useful for tracking the uptake and translocation of MPs in plants, as well as for studying their interactions with soil microbes.^38^ However, fluorescence microscopy requires the use of fluorescent MPs or the application of fluorescent dyes, which may not accurately represent the behavior of naturally occurring MPs.^38^

## 7. Effect of MPs on soil microbial communities: protists and nematodes in soil microbial dynamics

**Figure 2.**
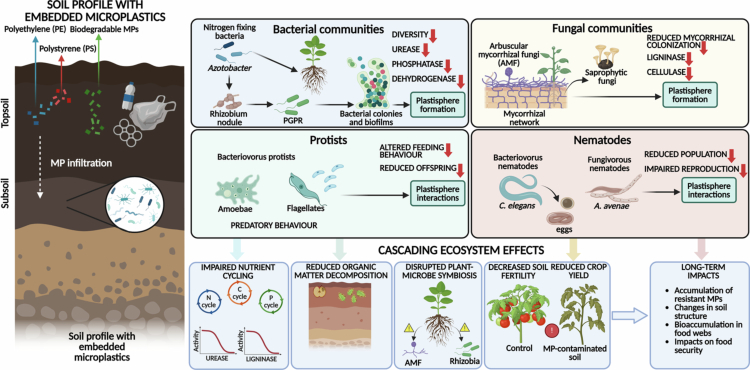
Impacts of MPs on soil microbial communities and ecosystem services. MPs, including PE, PS, and biodegradable plastics, infiltrate soil layers and interact with diverse soil biota. MPs influence bacterial communities by reducing diversity, enzymatic activities, and plant growth-promoting rhizobacteria, while enhancing plastisphere formation. Fungal communities exhibit reduced mycorrhizal colonization and ligninolytic and cellulolytic activities. Protists and nematodes show altered feeding behavior, impaired reproduction, and population decline following MP exposure. These disruptions collectively impair nutrient cycling, reduce organic matter decomposition, disturb plant–microbe symbiosis, and decrease soil fertility and crop productivity. Long-term accumulation of resistant MPs may further alter soil structure, microbial biodiversity, and food security, posing significant ecological and agricultural risks.

Soil microbial communities, including bacteria, fungi, protists, and nematodes, play essential roles in nutrient cycling, organic matter decomposition, and plant health (Figure 2). The presence of MPs in soil can disrupt these communities, with potential consequences for ecosystem functioning and agricultural productivity. The environmental fate and ecological impacts of biodegradable MPs on microbial communities are not yet fully understood. While biodegradable materials are designed to minimize long-term pollution, their degradation products, including MPs and chemical additives, may still pose risks to soil microbial health and functioning.^25^ Moreover, the presence of biodegradable MPs in soil can influence microbial community composition and activity, with potential consequences for nutrient cycling and ecosystem functioning.^32^ Biodegradable MPs can also serve as substrates for microbial colonization, potentially altering the structure and function of soil microbial communities. Some studies have reported that biodegradable plastics can stimulate microbial activity and enhance the degradation of organic matter, while others have found that they can inhibit microbial growth or promote the proliferation of specific microbial taxa.^32^ The interactions between biodegradable MPs and soil microbes are complex and depend on factors such as polymer composition, particle size, and environmental conditions.^24^

### 7.1. Effects on bacterial communities

Bacteria are the most abundant and diverse group of microorganisms in soil, contributing to a wide range of ecosystem processes, including nitrogen fixation, nitrification, denitrification, and organic matter decomposition.^39^ The presence of MPs in soil can alter bacterial community composition and activity, with effects that vary depending on MPs type, concentration, and environmental conditions.^32^ Studies have shown that MPs can reduce bacterial diversity and abundance, particularly for beneficial taxa such as nitrogen-fixing bacteria and plant growth-promoting rhizobacteria (PGPR).^32^ For example, exposure to PE MPs has been shown to reduce the abundance of Rhizobium and Azotobacter, two key nitrogen-fixing bacteria, in agricultural soils.^32^ Moreover, MPs can also influence bacterial activity and function. Studies have reported that MPs can inhibit the activity of key enzymes involved in nutrient cycling, such as urease, phosphatase, and dehydrogenase, potentially reducing the rates of nitrogen mineralization and phosphorus solubilization.^32^ These effects can have cascading impacts on plant productivity and soil fertility, particularly in nutrient-limited systems.^32^ Moreover, MPs can serve as substrates for the colonization of pathogenic bacteria, potentially increasing the risk of plant diseases and reducing crop yields (Figure 2).

### 7.2. Effects on fungal communities

Fungi are essential components of soil ecosystems, contributing to organic matter decomposition, nutrient cycling, and plant health through symbiotic relationships such as mycorrhizae^40^ (Figure 2). The presence of MPs in soil can disrupt fungal communities, with potential consequences for ecosystem functioning and agricultural productivity.^32^ Studies have shown that MPs can reduce fungal diversity and abundance, particularly for beneficial taxa such as arbuscular mycorrhizal fungi (AMF) and saprophytic fungi.^32^ For example, exposure to polystyrene (PS) MPs has been shown to reduce the colonization of plant roots by AMF, potentially impairing nutrient uptake and stress tolerance. In addition to their effects on fungal diversity, MPs can also influence fungal activity and function. Studies have reported that MPs can inhibit the production of extracellular enzymes involved in organic matter decomposition, such as cellulase, ligninase, and chitinase, potentially reducing the rates of carbon and nitrogen cycling.^32^ These effects can have cascading impacts on soil fertility and plant productivity, particularly in systems that rely on fungal-mediated nutrient cycling.^32^ Moreover, MPs can serve as substrates for the colonization of pathogenic fungi, potentially increasing the risk of plant diseases and reducing crop yields.^32^

### 7.3. Effects on protists and nematodes

Protists and nematodes are important components of the soil food web, contributing to nutrient cycling, organic matter decomposition, and the regulation of bacterial and fungal populations.^41^ The presence of MPs in soil can disrupt these communities, with potential consequences for ecosystem functioning and agricultural productivity.^32^ Studies have shown that MPs can reduce the abundance and diversity of protists and nematodes, particularly for bacterivorous and fungivorous taxa that play key roles in nutrient cycling.^32^ For example, exposure to PE MPs has been shown to reduce the abundance of amoebae and flagellates, two important groups of bacterivorous protists, in agricultural soils.^32^

In addition to their effects on protist and nematode diversity, MPs can also influence their activity and function (Figure 2). Studies have reported that MPs can alter the feeding behavior and reproduction of nematodes, potentially reducing their contribution to nutrient cycling and organic matter decomposition.^32^ These effects can have cascading impacts on soil fertility and plant productivity, particularly in systems that rely on protist- and nematode-mediated nutrient cycling.^32^ Moreover, MPs can serve as substrates for the colonization of pathogenic protists and nematodes, potentially increasing the risk of plant diseases and reducing crop yields.^32^

## 8. MPs uptake and translocation in plants

**Figure 3.**
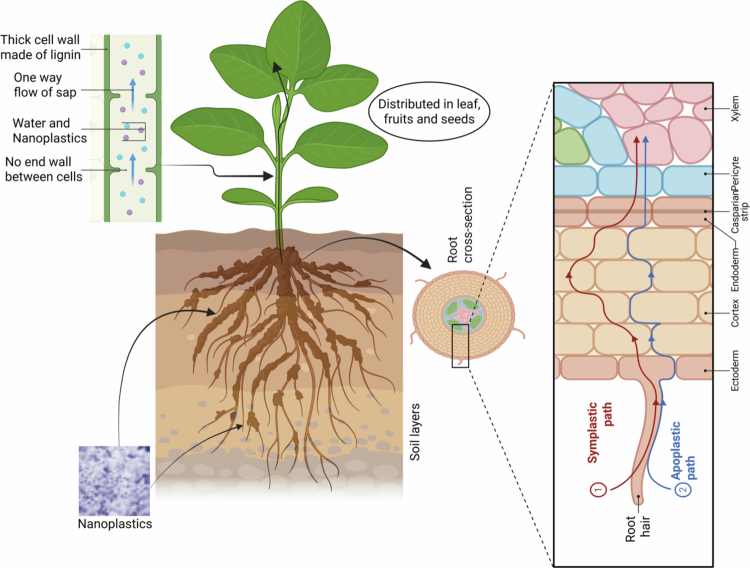
Schematic illustration of nanoplastics uptake, transport, and distribution in plants. Nanoplastics in soil are primarily absorbed through root hairs and enter root tissues via two major pathways: the apoplastic and symplastic pathways. The Casparian strip in the endodermis regulates particle movement into the vascular tissues. Once inside the xylem, particles are transported upward through transpiration-driven sap flow and distributed to aerial plant parts, including leaves, fruits, and seeds. The inset on the left shows xylem vessels with lignified thick cell walls that facilitate one-way transport of water, nutrients, and associated nanoplastics.

The uptake and translocation of MPs in plants have emerged as critical areas of research, given the potential for these particles to enter the food chain and affect human health. However, it is important to note that plastic uptake by plants is mainly limited to nanoplastics (particles <1 μm), not microplastics, due to size constraints at cellular and tissue levels. While larger MPs (1–5 mm) can adhere to root surfaces and affect plant physiology through external interactions, only nanoplastics can be internalized and translocated within plant tissues. Understanding the mechanisms and extent of nanoplastic uptake is essential for assessing the risks associated with MPs contamination in agricultural systems (Figure 3).

### 8.1. Mechanisms of nanoplastic uptake

Nanoplastics can be taken up by plant roots through several pathways, including apoplastic and symplastic routes.^42^ The apoplastic pathway involves the movement of particles through the cell walls and intercellular spaces, bypassing the plasma membrane.^42^ This pathway is generally accessible to particles smaller than 5–20 nm, depending on the plant species and the structure of the cell wall.^42^ The symplastic pathway, on the other hand, involves the uptake of particles through the plasma membrane and their subsequent movement through the cytoplasm and plasmodesmata.^42^ This pathway is more restrictive and typically limited to particles smaller than 50 nm.^42^ It is important to emphasize that microplastics (1–5 mm) are generally too large to be taken up through these pathways, and their effects on plants are primarily mediated through external interactions with root surfaces and the rhizosphere environment (Figure 3).

The uptake of nanoplastics by plant roots is influenced by several factors, including particle size, surface charge, and the presence of organic coatings or biofilms.^42^ Smaller particles are more readily taken up than larger ones, and particles with positive surface charges tend to be more efficiently internalized due to electrostatic interactions with the negatively charged cell membrane.^42^ The presence of organic coatings, such as humic acids or root exudates, can also enhance nanoplastic uptake by altering particle surface properties and reducing aggregation.^42^ Once inside the root, nanoplastics can be translocated to aerial parts of the plant through the xylem, the vascular tissue responsible for water and nutrient transport.^42^ The translocation of nanoplastics is driven by the transpiration stream and is influenced by factors such as particle size, plant species, and environmental conditions.^42^ Studies have shown that nanoplastics can accumulate in stems, leaves, and even reproductive tissues, raising concerns about their potential transfer into the food chain.^42^ However, larger microplastics are not translocated within plants and remain associated with root surfaces or the rhizosphere.

### 8.2. Evidence from experimental studies

Experimental studies have provided evidence for the uptake and translocation of nanoplastics in a variety of plant species, including crops such as wheat, lettuce, and tomato.^43^ ^,^ ^44^ For example, a study by Li et al.^43^ found that fluorescently labeled PS nanoplastics (100 nm) were taken up by wheat roots and translocated to shoots, with accumulation observed in both vascular and non-vascular tissues. Similarly, Sun et al.^44^ reported that lettuce plants exposed to PS nanoplastics (50 nm) accumulated these particles in roots, stems, and leaves, with higher concentrations observed in roots than in aerial tissues.

The uptake and translocation of nanoplastics have been shown to vary depending on particle size, with smaller particles being more readily taken up and translocated than larger ones.^43^ For example, a study by Lian et al.^42^ found that PS nanoplastics (50 nm) were more efficiently taken up by Arabidopsis roots than larger particles (500 nm), and that smaller particles were more readily translocated to shoots. These findings suggest that the size of nanoplastics is a critical factor determining their bioavailability and potential for accumulation in plant tissues.

### 8.3. Implications for food safety and human health

The uptake and translocation of nanoplastics in plants raised significant concerns about food safety and human health, as these particles can potentially enter the food chain through the consumption of contaminated crops.^43^ While the health effects of nanoplastic ingestion are not yet fully understood, studies in animal models have shown that these particles can cause oxidative stress, inflammation, and cellular damage, with potential implications for human health.^43^ Moreover, nanoplastics can serve as vectors for the transport of toxic chemicals, such as heavy metals and organic pollutants, which can be released into the body upon ingestion.^43^ The extent of nanoplastic contamination in food crops is not yet well characterized, and there is a pressing need for more research on the uptake, translocation, and accumulation of these particles in edible plant tissues.^43^ Developing standardized methods for detecting and quantifying nanoplastics in food is essential for assessing the risks associated with MPs contamination and for informing regulatory policies aimed at protecting public health.^43^ It is important to note that while nanoplastics pose potential risks through internalization and translocation, larger microplastics primarily affect plants through external mechanisms and are less likely to directly enter the food chain through plant uptake.

## 9. Hormetic and beneficial effects of MPs on plants

While the majority of research has focused on the detrimental effects of MPs on plant health, some studies have reported beneficial or hormetic effects at low concentrations. Hormesis refers to a biphasic dose-response relationship in which low doses of a stressor stimulate beneficial effects, while high doses cause toxicity.^45^ Understanding these context-dependent effects is essential for developing a comprehensive understanding of MPs impacts on plant physiology and for informing risk assessment and management strategies.

### 9.1. Enhanced root growth and biomass

Some studies have reported that low concentrations of MPs can enhance root growth and biomass in certain plant species, potentially due to improved soil aeration and water retention.^33^ ^,^ ^46^ The mechanisms underlying these beneficial effects are not fully understood but may involve changes in soil physical properties, such as increased porosity and reduced bulk density, which can facilitate root penetration and water infiltration.^46^ Additionally, MPs may alter the composition and activity of rhizosphere microbial communities, potentially enhancing the availability of nutrients or promoting the production of plant growth-promoting compounds.^46^

However, it is important to note that these beneficial effects are typically observed only at low MPs concentrations and may be offset by detrimental effects at higher concentrations.^46^ Moreover, the beneficial effects of MPs are highly context-dependent and may vary depending on factors such as plant species, soil type, and environmental conditions.^46^ Further research is needed to elucidate the mechanisms underlying these effects and to determine the conditions under which MPs may have beneficial rather than detrimental impacts on plant health.

### 9.2. Improved water retention and nutrient availability

In some cases, MPs have been reported to improve water retention and nutrient availability in soil, potentially benefiting plant growth under water-limited or nutrient-poor conditions^36^. For example, de Souza Machado et al.^36^ found that the addition of polyester fibers to soil increased water-holding capacity and reduced water stress in onion plants, with effects attributed to changes in soil structure and porosity. Similarly, some studies have reported that MPs can enhance the availability of certain nutrients, such as nitrogen and phosphorus, by altering soil pH or by serving as substrates for microbial colonization.^36^ However, these beneficial effects are not universally observed and may depend on factors such as MPs type, concentration, and soil properties.^36^ In many cases, MPs have been shown to reduce water retention and nutrient availability, particularly at high concentrations or in soils with low organic matter content.^36^ Moreover, the long-term effects of MPs on soil water and nutrient dynamics are not yet well understood, and there is a need for more research on the mechanisms underlying these effects and their implications for agricultural productivity.^36^

### 9.3. MPs as physical barriers

MPs can act as physical barriers in soil, influencing water movement, root growth, and microbial activity. The formation and effects of these barriers depend on soil structure, type, and texture, as well as on mechanisms such as bioturbation. The effects of MPs as physical barriers are complex and can be both beneficial and detrimental, depending on the context.^8^ For example, MPs can create pore spaces that enhance water infiltration and root penetration, potentially improving plant growth under certain conditions.^8^ However, at high concentrations, MPs can disrupt soil structure and reduce water retention, leading to increased water stress and reduced plant productivity.^8^

The role of MPs as physical barriers is also influenced by their size, shape, and distribution in the soil profile.^8^ Larger MPs, such as fragments and films, are more likely to create physical barriers that impede root growth and water movement, while smaller MPs, such as fibers and spheres, may have less pronounced effects.^8^ Moreover, the distribution of MPs in the soil profile, which is influenced by factors such as bioturbation and water movement, can determine the extent to which they act as physical barriers.^8^

## 10. Direct effects of MPs on plant physiology

**Figure 4.**
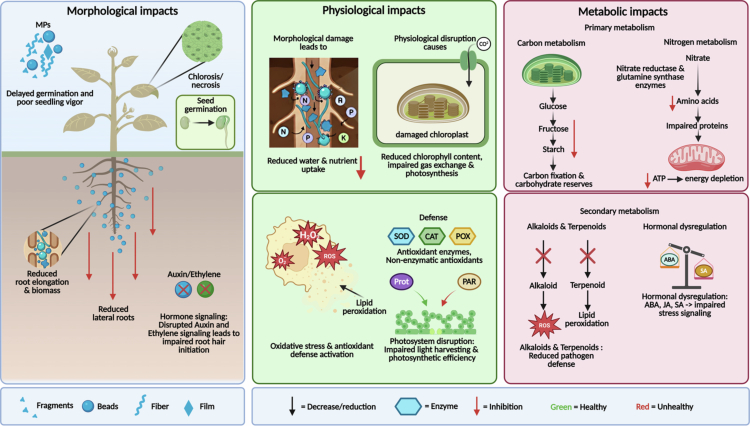
Effect of MPs on plant morphology, physiology, and metabolism. Morphological effects include delayed seed germination, reduced seedling vigor, chlorosis/necrosis, impaired root elongation, decreased biomass, reduced lateral root formation, and disruption of auxin–ethylene signaling pathways affecting root hair initiation. Physiological alterations involve reduced water and nutrient uptake, chloroplast damage, impaired gas exchange and photosynthesis, oxidative stress through excessive reactive oxygen species (ROS) generation, lipid peroxidation, and activation of antioxidant defense systems including superoxide dismutase (SOD), catalase (CAT), peroxidases (POX), proteases (Prot), and poly(ADP-ribose) polymerase (PAR). Metabolic disturbances include disruptions in carbon and nitrogen metabolism, reduced carbohydrate reserves, impaired amino acid and protein synthesis, ATP depletion, altered secondary metabolite biosynthesis (alkaloids and terpenoids), increased oxidative damage, and hormonal dysregulation involving abscisic acid, jasmonic acid, and salicylic acid. Different MPs forms, including fragments, beads, fibers, and films, are illustrated.

MPs can directly affect plant physiology through a variety of mechanisms, including physical obstruction, chemical leaching, and oxidative stress. These effects can manifest at different stages of plant development, from germination to maturity, and can have significant implications for crop productivity and food security (Figure 4).

### 10.1. Effects on seed germination

Seed germination is a critical stage in plant development, and exposure to MPs during this stage can have lasting effects on plant growth and productivity. Studies have shown that MPs can delay or inhibit seed germination in a variety of plant species, with effects that vary depending on MPs type, concentration, and particle size.^47^ ^,^ ^48^ For example, Bosker et al.^47^ found that exposure to PS MPs (1–5 μm) reduced germination rates in garden cress, with effects attributed to physical obstruction of water uptake and chemical leaching of toxic additives. Similarly, Machado et al.^48^ reported that exposure to PE MPs (10–45 μm) delayed germination in lettuce, with effects associated with reduced water absorption and impaired enzyme activity.

The mechanisms underlying MPs-induced inhibition of seed germination are not fully understood but may involve physical blockage of water and oxygen uptake, chemical toxicity from leached additives, and oxidative stress.^47^ MPs can adhere to seed surfaces, forming a physical barrier that impedes water imbibition and gas exchange, both of which are essential for germination.^47^ Additionally, MPs can leach toxic chemicals, such as plasticizers and stabilizers, which can interfere with enzyme activity and disrupt metabolic processes during germination.^47^

### 10.2. Effects on root growth and development

Root growth and development are essential for plant anchorage, water and nutrient uptake, and overall plant health. Exposure to MPs can impair root growth and development through a variety of mechanisms, including physical obstruction, chemical toxicity, and oxidative stress.^33^ ^,^ ^43^ Studies have shown that MPs can reduce root length, biomass, and lateral root formation in a variety of plant species, with effects that vary depending on MP type, concentration, and particle size.^33^ ^,^ ^43^ For example, Li et al.^43^ found that exposure to PS MPs (100 nm) reduced root length and biomass in wheat, with effects attributed to physical blockage of root pores and oxidative stress. Similarly, Qi et al.^33^ reported that exposure to PE MPs (100–500 μm) reduced lateral root formation in lettuce, with effects associated with disrupted hormone signaling and impaired cell division. The mechanisms underlying MPs-induced inhibition of root growth are complex and may involve multiple pathways. MPs can physically obstruct root pores and interfere with water and nutrient transport, leading to reduced root elongation and biomass.^43^ Additionally, MPs can leach toxic chemicals that interfere with hormone signaling and cell division, disrupting the normal development of root tissues.^43^ Moreover, MPs can induce oxidative stress by promoting the production of reactive oxygen species (ROS), which can damage cellular components and impair root function.^43^

### 10.3. Effects on shoot growth and leaf development

Shoot growth and leaf development are critical for photosynthesis, carbon assimilation, and overall plant productivity. Exposure to MPs can impair shoot growth and leaf development through a variety of mechanisms, including reduced nutrient uptake, impaired photosynthesis, and oxidative stress.^49^ ^,^ ^50^ Studies have shown that MPs can reduce shoot height, leaf area, and leaf biomass in a variety of plant species, with effects that vary depending on MPs type, concentration, and particle size.^49^ ^,^ ^50^ For example, Hu et al.^49^ found that exposure to PS MPs (100 nm) reduced shoot height and leaf area in maize, with effects attributed to reduced nutrient uptake and impaired photosynthesis. Similarly, Veloccia et al.^50^ reported that exposure to PE MPs (10–45 μm) reduced leaf biomass in lettuce, with effects associated with oxidative stress and chlorophyll degradation.

The mechanisms underlying MPs-induced inhibition of shoot growth and leaf development are complex and may involve multiple pathways. MPs can reduce nutrient uptake by physically obstructing root pores or by sorbing nutrients from the soil solution, leading to nutrient deficiencies that impair shoot growth.^49^ Additionally, MPs can impair photosynthesis by reducing chlorophyll content, disrupting stomatal function, or inducing oxidative stress, all of which can limit carbon assimilation and reduce shoot biomass.^49^ Moreover, MPs can leach toxic chemicals that interfere with hormone signaling and cell division, disrupting the normal development of shoot tissues.^49^

### 10.4. Effects on photosynthesis and chlorophyll content

Photosynthesis is the primary process by which plants convert light energy into chemical energy, and any disruption to this process can have significant implications for plant productivity and survival. Exposure to MPs can impair photosynthesis through a variety of mechanisms, including reduced chlorophyll content, disrupted stomatal function, and oxidative stress.^51^ ^,^ ^52^ Studies have shown that MPs can reduce photosynthetic rates and chlorophyll content in a variety of plant species, with effects that vary depending on MPs type, concentration, and particle size.^51^ ^,^ ^52^ For example, Lian et al.^51^ found that exposure to PS MPs (100 nm) reduced photosynthetic rates and chlorophyll content in Arabidopsis, with effects attributed to oxidative stress and chlorophyll degradation. Similarly, Sun et al.^52^ reported that exposure to PE MPs (10–45 μm) reduced photosynthetic rates in lettuce, with effects associated with disrupted stomatal function and reduced light use efficiency. The mechanisms underlying MPs-induced inhibition of photosynthesis are complex and may involve multiple pathways. MPs can reduce chlorophyll content by inducing oxidative stress, which can damage chloroplast membranes and degrade photosynthetic pigments.^51^ Additionally, MPs can disrupt stomatal function by altering water relations or by inducing hormonal changes, leading to reduced CO₂ uptake and impaired photosynthesis.^51^ Moreover, MPs can interfere with light absorption and energy transfer within the photosynthetic apparatus, reducing the efficiency of light use and limiting carbon assimilation.^51^

### 10.5. Effects on nutrient uptake and metabolism

Nutrient uptake and metabolism are essential for plant growth and development, and any disruption to these processes can have significant implications for plant productivity and survival. Exposure to MPs can impair nutrient uptake and metabolism through a variety of mechanisms, including physical obstruction of root pores, sorption of nutrients to MPs surfaces, and disruption of microbial-mediated nutrient cycling.^37^ ^,^ ^53^ Studies have shown that MPs can reduce the uptake of essential nutrients, including nitrogen, phosphorus, and potassium, across a variety of plant species, with effects that vary with MP type, concentration, and particle size.^37^ ^,^ ^53^ For example, Jia et al.^53^ found that exposure to PS MPs (100 nm) reduced nitrogen uptake and assimilation in wheat, with effects attributed to reduced expression of nitrate reductase and glutamine synthetase. Similarly, Rillig et al.^37^ found that exposure to PE MPs (10–45 μm) decreased phosphorus uptake in lettuce, potentially due to the adsorption of phosphate onto MP surfaces.

The mechanisms underlying MPs-induced inhibition of nutrient uptake and metabolism are complex and may involve multiple pathways. MPs can physically obstruct root pores and interfere with nutrient transport, leading to reduced nutrient uptake.^53^ Additionally, MPs can sorb nutrients from the soil solution, reducing their bioavailability to plants.^53^ Moreover, MPs can disrupt microbial-mediated nutrient cycling by altering microbial community composition and activity, leading to reduced rates of nitrogen mineralization and phosphorus solubilization.^53^

### 10.6. Oxidative stress and antioxidant responses

Oxidative stress, characterized by the overproduction of reactive oxygen species (ROS), is a common response to environmental stressors, including MPs exposure. ROS, such as superoxide radicals (O₂⁻), hydrogen peroxide (H₂O₂), and hydroxyl radicals (OH·), can damage cellular components, including lipids, proteins, and DNA, leading to impaired cellular function and reduced plant growth.^43^ ^,^ ^54^ Studies have shown that exposure to MPs can induce oxidative stress in a variety of plant species, with effects that vary depending on MPs type, concentration, and particle size.^43^ ^,^ ^54^ For example, Li et al.^43^ found that exposure to PS MPs (100 nm) increased ROS production and lipid peroxidation in wheat, with effects attributed to disrupted cellular redox balance. Similarly, Riaz et al.^54^ reported that exposure to PE MPs increased ROS production and oxidative damage in lettuce, with effects associated with impaired antioxidant enzyme activity.

Plants have evolved a variety of antioxidant defense mechanisms to mitigate oxidative stress, including enzymatic antioxidants such as superoxide dismutase (SOD), catalase (CAT), and peroxidase (POD), as well as non-enzymatic antioxidants such as ascorbic acid, glutathione, and phenolic compounds.^43^ Exposure to MPs can alter the activity of these antioxidant systems, with effects that vary depending on MP type, concentration, and plant species.^43^ In some cases, MPs exposure can enhance antioxidant enzyme activity, potentially providing protection against oxidative damage.^43^ However, at high MPs concentrations or under prolonged exposure, antioxidant defenses may be overwhelmed, leading to oxidative damage and reduced plant growth.^43^

## 11. Molecular and biochemical responses of plants to MPs

The molecular and biochemical responses of plants to MPs exposure are complex and involve a variety of signaling pathways, gene expression changes, and metabolic adjustments. Understanding these responses is essential for elucidating the mechanisms of MPs toxicity and for developing strategies to enhance plant tolerance to MPs contamination.

### 11.1. Gene expression and transcriptional responses

Exposure to MPs can induce significant changes in gene expression in plants, affecting a wide range of biological processes, including stress responses, hormone signaling, and metabolic pathways.^55^ ^,^ ^56^ Studies using transcriptomic approaches have identified hundreds to thousands of differentially expressed genes in plants exposed to MPs, with effects that vary depending on MPs type, concentration, and plant species.^55^ ^,^ ^56^ For example, Santini et al.^55^ found that exposure to PS MPs (100 nm) altered the expression of genes involved in oxidative stress responses, hormone signaling, and cell wall modification in Arabidopsis. Similarly, Yuan et al.^56^ reported that exposure to PE MPs altered the expression of genes involved in photosynthesis, nutrient metabolism, and secondary metabolite biosynthesis in lettuce.

The transcriptional responses to MPs exposure are mediated by a variety of transcription factors and signaling pathways, including those involved in stress responses, hormone signaling, and developmental processes.^55^ For example, exposure to MPs can activate transcription factors such as WRKY, MYB, and bZIP, which regulate the expression of genes involved in oxidative stress responses, pathogen defense, and secondary metabolite biosynthesis.^55^ Additionally, MPs exposure can alter the expression of genes involved in hormone signaling pathways, such as those for abscisic acid (ABA), jasmonic acid (JA), and salicylic acid (SA), which play key roles in stress responses and plant defense.^55^

### 11.2. Hormonal regulation and signaling

Plant hormones play critical roles in regulating growth, development, and stress responses, and exposure to MPs can disrupt hormonal balance and signaling pathways.^55^ Studies have shown that MPs exposure can alter the levels and signaling of key plant hormones, including auxins, cytokinins, gibberellins, abscisic acid (ABA), jasmonic acid (JA), and salicylic acid (SA).^55^ For example, Santini et al.^55^ found that exposure to PS MPs (100 nm) reduced auxin levels and disrupted auxin signaling in Arabidopsis, leading to impaired root growth and development. Similarly, other studies have reported that MPs exposure can increase ABA levels, potentially enhancing stress tolerance but also inhibiting growth.^55^

The disruption of hormonal balance and signaling by MPs can have cascading effects on plant growth and development. For example, reduced auxin levels can impair root elongation and lateral root formation, while increased ABA levels can inhibit shoot growth and reduce photosynthesis.^55^ Additionally, altered JA and SA signaling can affect plant defense responses, potentially increasing susceptibility to pathogens or altering the production of secondary metabolites.^55^ Understanding the hormonal responses to MPs exposure is essential for elucidating the mechanisms of MPs toxicity and for developing strategies to enhance plant tolerance to MPs contamination.

### 11.3. Metabolic adjustments and secondary metabolite production

Exposure to MPs can induce significant metabolic adjustments in plants, affecting primary metabolism (e.g., carbon and nitrogen metabolism) as well as secondary metabolism (e.g., production of phenolic compounds, alkaloids, and terpenoids).^56^ ^,^ ^57^ Studies using metabolomic approaches have identified hundreds of metabolites that are differentially regulated in plants exposed to MPs, with effects that vary depending on MPc type, concentration, and plant species.^56^ ^,^ ^57^ For example, Roy et al.^57^ found that exposure to PS MPs (100 nm) altered the levels of sugars, amino acids, and organic acids in Arabidopsis, with effects attributed to disrupted carbon and nitrogen metabolism. Similarly, Yuan et al.^56^ reported that exposure to PE MPs reduced the levels of phenolic compounds and flavonoids in lettuce, with effects associated with impaired secondary metabolite biosynthesis.

The metabolic adjustments induced by MPs exposure can have significant implications for plant growth, stress tolerance, and nutritional quality. For example, reduced levels of sugars and amino acids can limit energy availability and protein synthesis, leading to impaired growth and development.^57^ Additionally, reduced levels of secondary metabolites, such as phenolic compounds and flavonoids, can impair antioxidant defenses and increase susceptibility to oxidative stress and pathogen attack.^56^ Moreover, changes in secondary metabolite composition can affect the nutritional and medicinal value of crops, with potential implications for human health.^56^

### 11.4. Protein expression and post-translational modifications

Exposure to MPs can alter protein expression and post-translational modifications in plants, affecting a wide range of cellular processes, including metabolism, stress responses, and signal transduction.^43^ Studies using proteomic approaches have identified hundreds of proteins that are differentially expressed or modified in plants exposed to MPs, with effects that vary depending on the type of MPs, concentration, and plant species.^43^ For example, Li et al.^43^ found that exposure to PS MPs (100 nm) altered the expression of proteins involved in photosynthesis, carbon metabolism, and stress responses in wheat. Similarly, other studies have reported that MPs exposure can alter the phosphorylation, ubiquitination, and glycosylation of proteins, affecting their activity, stability, and localization.^43^ The changes in protein expression and post-translational modifications induced by MPs exposure can have significant implications for plant physiology and stress tolerance. For example, reduced expression of photosynthetic proteins can impair carbon assimilation and reduce plant growth, while altered expression of stress-responsive proteins can affect the ability of plants to cope with environmental stressors.^43^ Additionally, changes in post-translational modifications can affect protein function and stability, potentially altering metabolic pathways and signaling networks.^43^

## 12. Conclusion and future perspectives

MPs have emerged as significant environmental contaminants in agricultural soils, with complex, multifaceted effects on plant health, soil microbial communities, and ecosystem functioning. This review integrates current evidence on the sources, distribution, and impacts of MPs in the rhizosphere, highlighting both the direct and indirect mechanisms through which these particles influence plant physiology, microbial communities, and overall soil ecology. Key findings include the variable, yet often detrimental, effects of MPs on seed germination, root and shoot development, photosynthesis, nutrient uptake, and oxidative stress responses in plants. Additionally, MPs have been shown to disrupt soil microbial communities, reducing beneficial microbes, promoting pathogens, and interfering with enzymatic functions, thereby indirectly undermining soil fertility and crop productivity. Despite the growing body of research on MPs in terrestrial ecosystems, significant knowledge gaps remain. There is a pressing need for standardized methodologies for detecting and quantifying MPs in soil, as well as for assessing their toxicity and ecological impacts. Advances in analytical techniques, such as µ-CT, fluorescence microscopy, and spectroscopic methods, are essential for improving our understanding of MPs contamination and for developing effective mitigation strategies. Also, more research is needed on the molecular and biochemical mechanisms underlying MPs toxicity, including the roles of gene expression, hormonal signaling, and metabolic adjustments in plant responses to MPs exposure.

Future research should also focus on the long-term effects of MPs on soil health and ecosystem functioning, as well as on the potential for MPs to interact with other environmental stressors, such as climate change, nutrient pollution, and pathogen pressure. Understanding these interactions is essential for predicting the broader ecological impacts of MPs contamination and for developing integrated management strategies that address multiple stressors simultaneously. Moreover, there is a need for more research on the fate and behavior of biodegradable MPs in agricultural soils, as these materials are increasingly being promoted as sustainable alternatives to conventional plastics. Recent studies have provided important insights into the effects of biodegradable and conventional mulching film MPs on soil properties, microbial functions, and plant performance across different environmental conditions, but further research is needed to fully understand their long-term ecological impacts.

## Data Availability

Data sharing does not apply to this article as no new data were created or analyzed in this study.

## Abbreviations

MPs: Microplastics
POPs: Persistent organic pollutants
PE: Polyethylene
PP: Polypropylene
PS: Polystyrene
PET: Polyethylene terephthalate
DOC: Dissolved organic carbon
ROS: Reactive oxygen species
